# Assembly and Annotation of Transcriptome Provided Evidence of miRNA Mobility between Wheat and Wheat Stem Sawfly

**DOI:** 10.3389/fpls.2017.01653

**Published:** 2017-09-26

**Authors:** Halise B. Cagirici, Sezgi Biyiklioglu, Hikmet Budak

**Affiliations:** ^1^Molecular Biology, Genetics and Bioengineering Program, Faculty of Engineering and Natural Sciences, Sabanci University, Istanbul, Turkey; ^2^Cereal Genomics Lab, Department of Plant Sciences and Plant Pathology, Montana State University, Bozeman, MT, United States

**Keywords:** WSS, larva, lncRNA, miRNA, interaction networks, tRNA, Wheat Stem Sawfly

## Abstract

Wheat Stem Sawfly (WSS), *Cephus Cinctus* Norton (Hymenoptera: Cephidae), is one of the most important pests, causing yield and economic losses in wheat and barley. The lack of information about molecular mechanisms of WSS for defeating plant’s resistance prevents application of effective pest control strategies therefore, it is essential to identify the genes and their regulators behind WSS infestations. Long non-coding RNAs (lncRNAs) and microRNAs (miRNAs) are recognized with their regulatory functions on gene expression, tuning protein production by controlling transcriptional and post-transcriptional activities. A transcriptome-guided approach was followed in order to identify miRNAs, lncRNAs, and mRNA of WSS, and their interaction networks. A total of 1,893 were presented here as differentially expressed between larva and adult WSS insects. There were 11 miRNA families detected in WSS transcriptome. Together with the annotation of 1,251 novel mRNAs, the amount of genetic information available for WSS was expanded. The network between WSS miRNAs, lncRNAs, and mRNAs suggested miRNA-mediated regulatory roles of lncRNAs as competing endogenous RNAs. In the light of the previous evidence that small RNA molecules of a pathogen could suppress the immune response of host plant, we analyzed the putative interactions between larvae and wheat at the miRNA level. Overall, this study provides a profile of larva and adult WSS life stages in terms of coding and non-coding elements. These findings also emphasize the potential roles of wheat and larval miRNAs in wheat resistance to infestation and in the suppression of resistance which is critical for the development of effective pest control strategies.

## Introduction

Wheat Stem Sawfly (WSS), *Cephus Cinctus* Norton (Hymenoptera: Cephidae) is stated as the most damaging pest of wheat in Northern Great Plains, causing crop devastations in Montana region each year (Beres et al., 2011). Female WSS choose the internodes of actively elongating fresh wheat stems to lay their eggs. By tearing the stem with their sharp ovipositors, eggs are placed into the stem where the larvae form after 4–7 days of incubation (Cárcamo et al., 2011). Since the larvae are cannibalistic, only one larva can survive in the stem although there are more eggs deposited. Larva stays and develops in the wheat stem during the growing season, feeding on parenchyma and vascular tissues and, eventually, it moves toward the bottom of the stem to cut a notch, causing plant to lodge in order to overwinter there until the pupation occurs. Stem cutting causes a dramatic reduction in yield, and even uncut infested plants have low yield due to decreased head weight by 17% (Delaney et al., 2010). However, there are still no effective control method over WSS damage in wheat. Usage of chemicals is limited by the long emergence period of females and the wheat stem protecting the eggs and the larva feeding inside (Knodel et al., 2009). The introduction of solid-stemmed wheat instead of hollow-stemmed wheat maintained a more powerful control on the infestations. Yet, the solid-stemmed cultivars are not preferred by producers because of its low yield and protein content compared to hollow-stemmed cultivars (Beres et al., 2011).

Until recent years, non-coding RNAs (ncRNAs) had been overshadowed by the interest on protein-coding RNAs and their pathways. As bioinformatics tools and experimental technologies brought new aspects in our understanding of RNA world, the structures and regulatory functions of ncRNAs came to light and most of the recent studies extended their focuses on microRNAs (miRNAs) and long non-coding RNAs (lncRNAs) (Charon et al., 2010; Lucas and Budak, 2012; Akpinar et al., 2015; Alptekin et al., 2016a). Both plant and animal miRNAs are ∼22 nucleotide-long molecules and are derived from transcripts that fold on themselves to form stem-loop structures. In animals, the primary sequences transcribed by RNA polymerase II are processed by Drosha and Dicer-1 enzymes to produce pre-miRNAs and finally, mature miRNA/miRNA^∗^ duplexes (Bartel, 2009). In plants, both processes are performed by Dicer-like protein (DCL) since plants lack Drosha enzyme (Budak and Akpinar, 2015). Upon unwinding of the duplex, mature miRNA is exposed to RNA-Induced Silencing Complex (RISC) to recruit them toward its target (Budak and Akpinar, 2015). miRNAs can bind their target mRNAs from either 3′ or 5′ UTR regions, with an imperfect complementarity (Bartel, 2009; Budak and Akpinar, 2015), resulting in transitional repression or degradation of the target (Alptekin et al., 2016b). The interactions between mature miRNAs and their target mRNAs provide an additional control on gene expression regulation. The first miRNA reported, lin-4, was shown to regulate timing of development through targeting lin-14 mRNA in *Caenorhabditis elegans* (He and Hannon, 2004; Alvarez-Garcia and Miska, 2005). Since then, distinct roles have been characterized for a vast number of miRNAs from animals and plants. Functional characterization of miRNAs in insect species have revealed the importance of miRNAs in several regulatory processes, including metabolism (Lucas and Raikhel, 2013), growth and development (Bilak et al., 2014), survival (Jones et al., 2013). miRNAs from one species may function at interspecies level, targeting genes or genomes of organisms which they have physical contact. Very recently, independent studies have been reported several examples of *trans*-kingdom delivery of sRNAs from; plant to virion (Iqbal et al., 2017), oomycetes to plant (Jia et al., 2017), plant to nematodes (Tian et al., 2016). Similar to what these studies suggested, miRNAs might also be effective in regulating insect–host interactions at WSS larval stages once larva gets into the stem of the host plant.

As being another important class of ncRNAs, lncRNAs draw attention with their mRNA-like structural features and biogenesis processes. Like mRNAs, they are expected to be longer than 200 nucleotides, subjected to alternative splicing and 5′ capping, and mainly transcribed by RNA polymerase II (Legeai and Derrien, 2015). None-to-very low coding-potential of lncRNAs is the major factor to differentiate lncRNAs from mRNAs. Several remarkable features of lncRNAs include the tendency to exhibit tissue and sample specific expressions (reviewed in Quinn and Chang, 2015), which can be speculated to the importance of lncRNAs in regulatory mechanisms. It has been shown that lncRNAs are indeed involved in key regulatory mechanisms across diverse biological processes, such as dosage compensation (Militti et al., 2014), developmental- and epigenetic- regulation (Schmitz et al., 2016) in various species. For example, a yellow-achaete intergenic RNA (yar) was found to be an effective component of the sleep behavior in *Drosophila melanogaster* (Soshnev et al., 2011). *D. melanogaster*, as a model organism, has been extensively investigated for its lncRNA genes (Soshnev et al., 2011; Li et al., 2012), although functions of the majority of lncRNAs in flies remain unknown (Xiao et al., 2015).

The interactions between miRNAs and lncRNAs are also critical for the regulation of gene expression since lncRNAs might act as miRNA precursors or miRNA targets. By binding on the complementary sites on the target lncRNAs, miRNAs decrease the stability of the target, controlling their abundance and regulatory function in the cell (Yoon et al., 2014). miRNAs and lncRNAs are both known to form decoys, titrating the transcription factors from the environment (Wang and Chang, 2011; Banks et al., 2012). Moreover, lncRNAs can function as endogenous Target mimics (eTMs) of miRNAs (Franco-Zorrilla et al., 2007) or competing endogenous RNAs (ceRNAs) (Salmena et al., 2011) of mRNAs where the target lncRNA titrates the miRNA to inhibit its pairing with the target mRNA.

In this study, transcriptome data from eight WSS samples were utilized to generate the assembly and, later, to identify miRNA, lncRNA, and mRNA molecules from larvae, female and male WSS. In total, we obtained 11 miRNA families, 40,185 coding transcripts and 59,676 lncRNA transcripts from the WSS transcriptome. Additionally, we constructed differential expression library of WSS transcripts to compare expression profiles of larva and adult WSS samples. Annotations and the expression profiles of transcripts will be useful resources in the understanding of the molecular mechanisms of WSS. Considering the effect of WSS larvae on wheat, we have focused on the action mechanisms of RNAs in larvae and their targets in wheat, and compared them with female and male adult data. Understanding the role of RNAs in infestation of wheat crop fields by WSS will give insight for future strategies in fighting with the pests and increasing the wheat yield.

## Materials and Methods

### *De Novo* Assembly and Differential Expression of Transcripts

RNA-Sequencing (RNA-Seq) of eight WSS samples (larvae, antennae, female, and male) was obtained from NCBI SR database [**Supplementary Table S1**; Sequence Read Archive (SRA) accession number SRP067708]. Trimmomatic (v0.32) with default parameters (LEADING:5, TRAILING:5, MINLEN:36) was used for adaptor trimming and quality trimming of reads (Bolger et al., 2014). A single assembly containing reads from all eight WSS samples was generated *de novo* using Trinity software (release 2014-07-17) (Grabherr et al., 2013). All transcripts were restricted to be >200 bp in length.

Trimmed raw reads were aligned back to the assembled transcripts using Bowtie assembler and abundance estimates of transcripts were quantified as Fragment Per Kilobase Million mapped reads (FPKM) using RSEM (version 3.2) (Li and Dewey, 2011) under Trinity pipeline. Differential expression analysis was performed using EdgeR (Robinson et al., 2010) pipeline with the default threshold parameters of *p*-value = 0,001 and log2 (fold_change) = 2. Assembly files of larvae, female, and male pooled whole samples were separated based on their corresponding abundance estimates for further analyses.

### Annotation of Transcripts and lncRNAs

Annotation of transcripts were performed by analyzing the reads in a four-step process; eliminating contaminants, separation by ORF size criteria, coding potential calculations and homology-based predictions. All assembled transcripts were aligned with known small non-coding RNA sequences of all hexapoda species deposited in NCBI (1711 sequences) using blastn (-*e*-value 1E-05). Since the focus was on the coding and lncRNA sequences, transcripts with homology to small non-coding RNAs were defined as contaminants and eliminated. The abilities of transcripts to code for a full-length protein was evaluated using Transdecoder under Trinity software. Transcripts with predicted open reading frames (ORFs) longer than 100 amino acids passed the ORF size criteria for annotation process. Coding potentials of the transcripts were calculated using two prediction techniques; CPC (online version, reverse strand included, 2016) (Kong et al., 2007) and CNCI (-s ve) (Sun et al., 2013). Transcripts predicted as ‘coding’ by at least one of these tools were accepted to be satisfied the coding potential prediction criteria. Homology-based predictions were performed through homology screenings against functional coding sequences using Blast (version 2.2.26) and against known protein domains with Pfam identification using Hmmer (v.3.1b1) (Zhang and Wood, 2003). All assembled transcripts were screened for homology to known mRNA sequences of WSS, protein sequences of *Cephus*, *Apis*, *Hymenoptera* families and Swissprot entries (all deposited at NCBI) using blast (-*e*-value 1E-05, -length 90, -identity 80). Peptide sequences of assembled transcripts with an ORF size longer than 30 amino acids were predicted using Transdecoder. These peptide sequences were further screened using blastp against Swissprot entries (1E-05, -length 30, -identity 80) and using Hmmer (v.3.1b1) against Pfam domains (1E-05). Transcripts with a homology to functional sequences or a predicted Pfam domain passed the homology-based prediction criteria.

Following this multi-layered analysis, putative coding transcripts were identified by excluding contaminant transcripts and selecting transcripts that passed ORF size, coding potential prediction and homology-based prediction analyses. On the other hand, knowing that lncRNAs do not possess open reading frames or protein-coding potentials, transcripts which failed in all homology-based, coding potential and ORF size prediction analyses were identified as putative lncRNAs. Actively-expressed transcripts were extracted according to the fpkm threshold of 0.5. Differential-expression analysis was performed through pair-wise comparison of sample-specific expressions of each transcript using edgeR software with *p*-value of 0.001 and fold-change of four thresholds. Actively-expressed mRNA and lncRNA transcripts were provided in **Supplementary Data S1**, **S2**.

### Identification and Annotation of miRNAs and tRNAs

High confidence mature miRNA sequences of hexapoda species were retrieved from miRBase database (v21, June 2016) (Kozomara and Griffiths-Jones, 2011). *In silico* miRNA prediction was performed based on homology and secondary structure predictions, as previously defined (Lucas and Budak, 2012; Kurtoglu et al., 2014) using this set of 562 mature miRNA sequences. In general, *de novo* assembled transcriptome was subjected to homology screening to predict putative mature miRNA sequences, allowing at most 1 base mismatch. Predicted mature miRNA sequences were extended from both ends to predict pre-miRNA sequences after when they can be subjected to UNAFold (Markham and Zuker, 2008) to simulate RNA folding. Secondary structure predictions evaluate characteristics of hairpin structure to differentiate miRNAs from other ssRNAs by several parameters including MFEI and GC content. Later, final evaluations were performed based on strict criteria of correct folding: (1) max number of mismatches allowed are 4 for miRNA and 6 for miRNA^∗^ sequences; (2) no mismatches allowed at Dicer-Like enzyme cut sites; (3) multi-loop structures are not allowed between miRNA and miRNA^∗^; (4) miRNA or miRNA^∗^ sequences cannot be involved in the head part of the hairpin, using in-house python scripts.

The genes encoding tRNA species were extracted using the local version of tRNAscan-SE software (Lowe and Eddy, 1996) with the default parameters for eukaryotic genomes.

### Prediction of miRNA Targets

Target transcripts of newly identified miRNAs were predicted using two algorithms, RNAhybrid (Krüger and Rehmsmeier, 2006) and miRanda (Enright et al., 2003). Filtering criteria were applied to each prediction as follows: RNAhybrid: *p*-value adjusted to 3utr_fly, mfe <= -25 kcal/mol; miRanda: total score > = 140, total energy <= -25 kcal/mol. Putative target transcripts were accepted from those predicted by the two software. The resulting putative mRNA targets were aligned to NCBI non-redundant (nr) protein database (blastx, -*e*-value 10-5, -outfmt 5) where blast top hits were functionally annotated using Blast2GO software. A list of target transcripts from lncRNAs and mRNAs targeted by the same mature miRNA sequences was gathered together to construct an interaction network between lncRNAs, miRNAs, and mRNAs, which was visualized using Cytoscape 3.3.0 (Shannon et al., 2003).

Identified larval mature miRNA sequences of WSS were further evaluated for their putative mRNA targets within wheat coding sequences by using psRNATarget online tool (Dai and Zhao, 2011). Functional annotation of the target mRNA sequences was performed using Blast2GO software following homology screening against protein sequences of 72 *Viridiplantae* species (blastx, -*e*-value 10-5, -outfmt 5, -max_target_seq 1).

## Results

### *De Novo* Assembly of WSS Transcriptome

RNA-sequencing data from eight WSS samples, including larvae, antennae, females, and males, from infected plants were retrieved from NCBI database [Sequence Read Archive (SRA) accession number SRP067708]. Initially, all reads were subjected to adaptor and quality trimming using Trimmomatic, revealing a total of 28.799 Gbp clean reads. Despite reducing the number of reads, this step improved the quality and the process time of the assembly. All trimmed reads were then assembled into one assembly using Trinity *de novo* assembler, resulting in 165,284 transcripts with a N50 length of 3,304 bases (**Table 1**), indicating the high-quality of the transcripts that could construct full-length protein sequences. GC content of the assembly was 40.65%, which is similar to the GC content of the raw reads (39–43%). A detailed summary of the assembly statistics can be found in **Table 1**. Clean raw reads were aligned back to the assembly to determine the expression levels of each transcript, which were scaled to fragment per kilobase million (fpkm). Based on the normalized fpkm values greater than 0.5 in at least one of the eight WSS samples, 143,483 (86.8%) transcripts were defined as actively-expressed WSS transcripts.

**Table 1:** Summary statistics of sequencing and combined *de novo* transcriptome assembly of Wheat Stem Sawfly (WSS).

**Read processing**	
Reads before trimming	50.248 gb
Reads after trimming	28.799 gb
**Assembly statistics**	
Number of ‘genes’	116560
Number of transcripts	165284
Percent GC	40.65
N50 (bp)	3304
Median contig length	523
Average contig	1380.63
Total assembled bases	228196136

### Annotation of WSS Transcriptome

To elucidate interactions of non-coding RNAs (lncRNAs and miRNAs) with protein-coding sequence content of WSS, all actively expressed transcripts were subjected to a selection process, following the transcriptome assembly. Transcripts satisfying the criteria of having homology to known coding sequences, a predicted coding potential and an ORF region that is at least 100 amino acid-long were defined as candidate mRNA transcripts (called mRNA transcripts from now on). Thus, 40,185 mRNA transcripts were identified, of which 38,934 (96.86%) of them showed significant resemblance to known WSS mRNAs with 80% or more identity (**Supplementary Table S2**), indicating 1,251 novel mRNAs were identified. These novel mRNAs were screened through NCBI non-redundant (nr) protein database for similarity to a known protein from other organisms, thereby revealing potential functions of transcripts. Functions of proteins with significant hits included tRNA ligases, histone proteins, kinases and more (**Supplementary Table S3**). Although all novel mRNAs showed significant homology to at least one known protein, only 868 of them were mapped to 15,947 Gene Ontology (GO) terms. These GO terms represented molecular functions (MF) of newly identified mRNAs as binding, catalytic activity and structural molecule activity where their biological processes (BP) were predicted as metabolic, cellular or single-organism processes at level 2. At a multi-level classification, ion binding and biosynthetic process were the most predominant annotations in the MF and BP categories, respectively.

**FIGURE 1:**
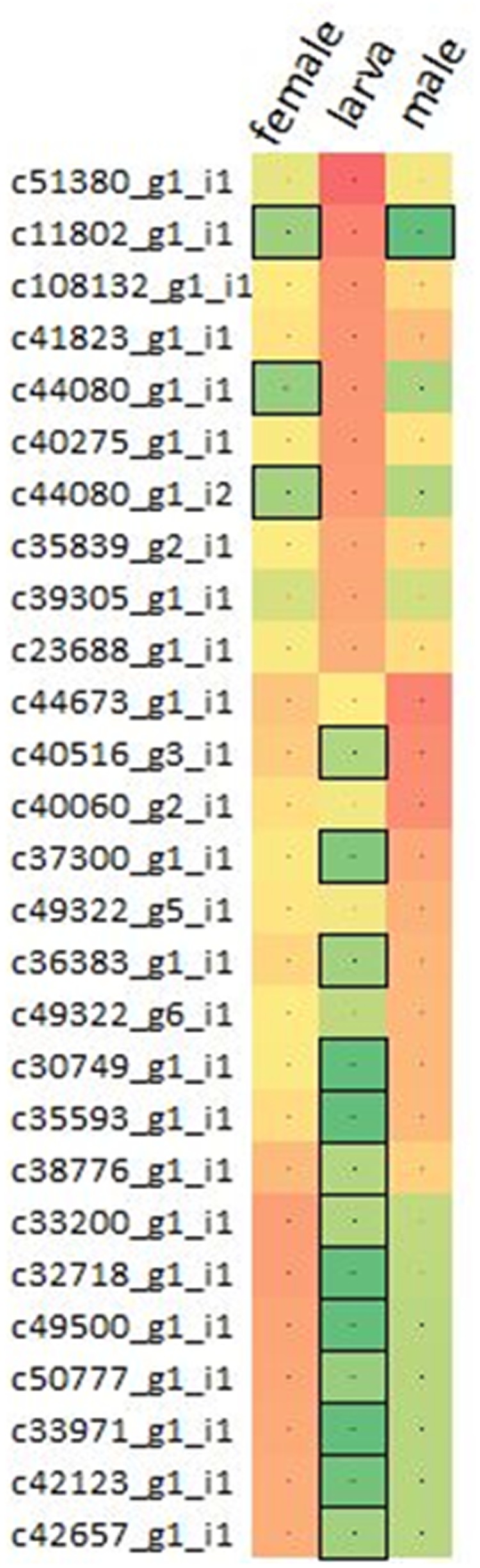
Comparison of the expressions of transcripts. Top 10 differentially expressed transcripts with the highest expressions were collected from pooled larva, male and female samples, totaling 27 non-redundant list of transcripts. Expressions were presented in terms of log10(fpkm) from red to green, representing high to low expression. Transcripts having low-to-none expressions (<2fpkm) were highlighted with the boxes.

Varying sets of expressed mRNA transcripts showed differential expression between larva and adult WSS samples, reflecting the effect of developmental stage on the WSS transcriptome. Differential-expression analysis performed through pair-wise comparison of sample-specific expressions of each transcript revealed 16,291 and 16,928 mRNAs that were differentially-expressed between larva-adult male and larva-adult female samples, respectively, where 12,453 of them were common in both comparison pairs, totaling 20,766 mRNAs differentially expressed between larva and male or female samples. A list of differentially expressed transcripts has been compiled combining ten transcripts with the highest levels of expression from each of the larva, male and female samples. Three of the top 10 highly expressed transcripts of female and male samples coincided, totaling 27 differentially expressed transcripts with the top 10 highest levels of expression in one of the three samples (**Figure 1**).

**FIGURE 2:**
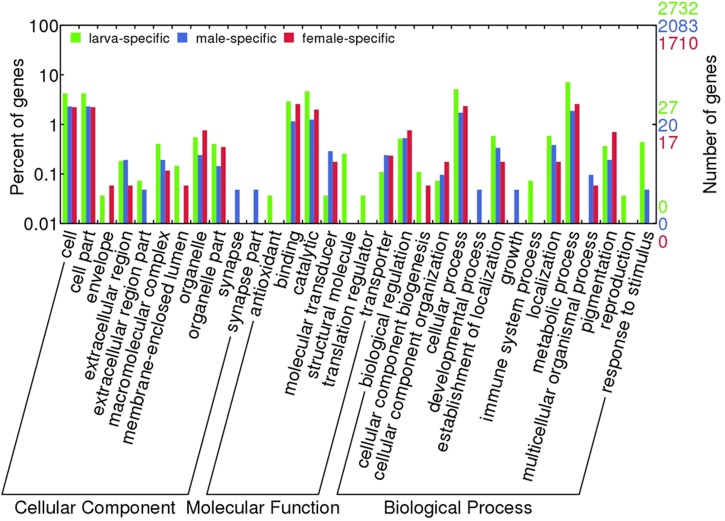
Blast2GO term distribution over differentially expressed transcripts. Transcripts with sample specific expressions were shown.

Comparative functional annotation of mRNA transcripts revealed that 2,732, 2,083, and 1,710 transcripts were exclusively expressed in larva, male and female samples, respectively. These mRNA transcripts were composed of proteins known to be involved in various biological processes (**Figure 2**), which exclusively were in immune system process and reproduction in larva, and developmental process and growth in males. Besides, antioxidant and translation regulation MFs were identified only in larva samples. Unfortunately, hypothetical, predicted and unknown proteins made up to 25% of these transcripts, which points out to that there might be many additional pathways that these differentially expressed transcripts play roles in.

### Identification of lncRNAs

The analyses for lncRNA identification yielded a total of 71,220 putative lncRNAs, which corresponded to 4.09% of all transcripts of the Trinity-assembled transcriptome of WSS. Based on normalized fpkm which was greater than 0.5 in at least one of the eight WSS samples, actively-expressed lncRNA transcripts (named as lncRNAs from this point) were identified for further analyses. The results showed that 83.79% (59,676) of lncRNAs passed the threshold of active expression as opposed to 92.21% (40,185 out of 43,581) of annotated transcripts, illustrating the tendency of lncRNAs to exhibit lower expressions.

**FIGURE 3:**
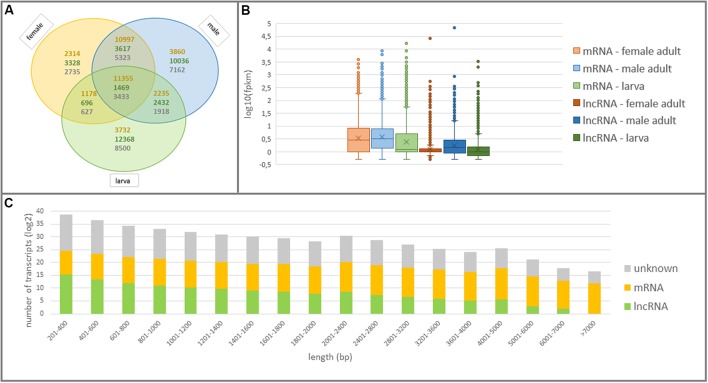
Structural features of coding and non-coding elements in WSS transcriptome. **(A)** Venn diagram shows the numbers of common and specific elements in larva, male and female WSS samples. The numbers of mRNAs, lncRNAs and unknown transcripts were written in orange, green, and gray colors, respectively. **(B)** The expression patterns of mRNAs and lncRNAs in larva, male and female samples. **(C)** Length distribution of the transcripts expressed in any WSS samples.

All lncRNAs were further examined in terms of expression patterns in larva and adult WSS samples to discover larva-specific and adult-specific lncRNAs in WSS. Among a total of 59,676 actively-expressed lncRNA transcripts, 55,946 (56.88%) of them possessed a normalized fpkm greater than 0.5 in at least one of the larva, male or female WSS samples. It appeared that lncRNAs were the most abundant in larva followed by male and female WSS transcriptomes. 16,965 (34%) of 49,943 actively-expressed larva transcripts were defined as lncRNAs as opposed to 17,554 (27%) of 63,837 male transcripts and 9,110 (19%) of 47,042 female transcripts (**Figure 3A**). Moreover, most of the larva and male lncRNAs were sample-specific whereas most of the female lncRNAs were common in either one of the samples. This comparison of lncRNA content of the three samples indicated that larva showed the highest and female the lowest, transcriptional diversity and specificity. These results suggested the abundance of lncRNAs in larvae compared to adult WS, indicating the functional importance of lncRNAs in different levels of WSS life cycle, especially in the larval stages.

mRNA transcripts, on the other hand, showed less sample-specific expressions than lncRNA transcripts. In fact, 88.77% (35,671) of actively-expressed mRNA transcripts exhibited expression evidence in at least two of the larva, male and female samples as opposed to 56.88% of actively-expressed lncRNA transcripts (**Figure 3A**). Besides, 31.83% of these mRNAs were common in all three WSS samples and 66.62% of them were shared by more than one samples whereas that of 2.63% of common lncRNAs and 14.68% of shared lncRNAs. Further examination of expression levels of lncRNA and mRNA transcripts showed lower levels of lncRNA expression in all three WSS samples (**Figure 3B**). These results indicated sample-specific expression patterns as well as lower expression levels of lncRNAs than of mRNAs.

To determine lncRNAs that were either upregulated, downregulated or showed no differential expression between different WSS samples, a pairwise differential expression analysis was performed using edgeR package under Trinity software. It was found that 1,893 of the lncRNAs were differentially expressed between larva and adult WSS samples. 728 of those differentially expressed lncRNAs were upregulated in larva samples whereas 686 and 1,059 of them showed upregulation in female and male adult samples when compared to larva. Although there were more sample specific lncRNAs identified, these differentially expressed lncRNAs were the ones that passed the strict criteria.

### Characteristics of lncRNAs and mRNAs

We analyzed structural features of all actively-expressed lncRNA transcripts and compared with the ones for mRNA transcripts in WSS. The lengths of the lncRNAs ranged from 201 to 6,465 bp. Most of the lncRNAs, however, had shorter transcripts such that 93.6% of the lncRNAs were shorter than 1,000 bp (**Figure 3C**). On the other hand, mRNA transcripts were remarked by longer sequences such that longest mRNA transcripts contained 27,058 nt and half of them were longer than 2,990 nt. Average transcript length of lncRNAs was 444 bp as opposed to that of 3614 bp for mRNA transcripts. In addition, GC contents were ranging between 8 and 70% for lncRNAs and 26 to 72% for mRNA transcript, the majority of which (83 and 91% for lncRNA and mRNA transcripts, respectively) were around 30 to 50% (**Supplementary Figure S1**). Average GC content for lncRNA and mRNA transcripts were 39 and 42%, respectively. The longest transcripts, of both lncRNAs and mRNAs, were the ones with average GC content. We could not detect any significant correlation between length and GC content of both mRNA and lncRNA transcripts.

Alternative splicing is one of the common features between lncRNAs and mRNAs although lncRNAs have lower splicing ratio than protein-coding genes in mammals. Consistent with their counterparts in the mammals, WSS lncRNAs showed less splicing than annotated transcripts. Alternatively-spliced isoforms were identified for only 11% (6,376) of the lncRNA transcripts in this assembly, which is significantly lower than 83% (33,537) of the ratio observed in annotated transcripts. Among the lncRNAs having alternatively spliced isoforms, 20% (1,286) of them shared at least 4 isoforms, which is less than one third the ratio of 69% (23,079) for mRNA transcripts having alternatively spliced isoforms. Such low levels of splicing events in lncRNA transcripts indicated that it is not as common as in mRNA transcripts of WSS. As an exception, 76 of the putative lncRNAs showed high splicing events with at least twelve isoforms. The maximum number of alternative splicing in lncRNAs was 23, observed in the gene, c49416_g1. Five isoforms of this gene were identified as putative lncRNAs. Two of these lncRNAs failed to pass expression threshold in larva, male, female WSS samples. Remaining lncRNAs exhibited sample specific expressions where c49416_g1_i22 expressed only in male, and c49416_g1_i23 and c49416_g1_i6 expressed only in larva samples. These estimated abundances of transcripts over different samples revealed the unique expression profiles of the alternatively spliced isoforms in the different stages of WSS life cycle.

### tRNA Annotation

**FIGURE 4:**
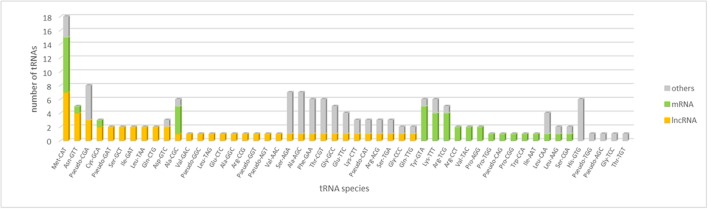
tRNA content of mRNA, lncRNA and the remaining transcripts in the WSS transcriptome. tRNA species sorted by their abundance in lncRNAs, mRNAs and others in order.

The analysis of tRNA gene content of WSS transcriptome revealed that the majority of tRNA gene families were represented by more than a single copy in the WSS transcriptome. A total of 159 putative tRNA genes were identified, 41 and 50 of which were encoded by actively-expressed mRNA and lncRNA transcripts, respectively (**Figure 4**). These tRNA genes correspond to 21 putative tRNA gene families with a specificity for 45 anticodons. With a total of 18 loci, tRNA:Met-CAT was marked as the most abundant tRNA species among all WSS transcriptome as well as among mRNA (8) and lncRNA (7) transcripts. The codon it decodes, AUG, is the most common canonical start codon. Moreover, several tRNA species were encoded by only mRNAs or lncRNAs but not by any other transcripts. Eight and 14 tRNA species were found to be either mRNA or lncRNA specific, respectively. For the remaining tRNA species, we could not detect any correlation between mRNA and lncRNA transcripts.

### *In Silico* miRNA Prediction

Using 562 high confidence mature miRNA sequences from hexapoda species deposited at miRbase, a total of 18 mature miRNA sequences referring to 11 miRNA families were identified from the assembly of WSS transcriptome. Among these miRNA families, four miRNA families, miR-281 (4), miR-8 (3), miR-10 (2), and miR-14 (2), were represented with more than one stem-loops (**Supplementary Table S4**). Predicted mature miRNA and pre-miRNA sequences were ranging between 21–23 nt and 94–125 nt, respectively. Average length of all putative mature miRNA sequences was 22 nt where that of 99 nt for their respective pre-miRNA sequences. These values are consistent with the 80–100 nt mean sequence length of animal miRNAs (Greenberg et al., 2012).

**FIGURE 5:**
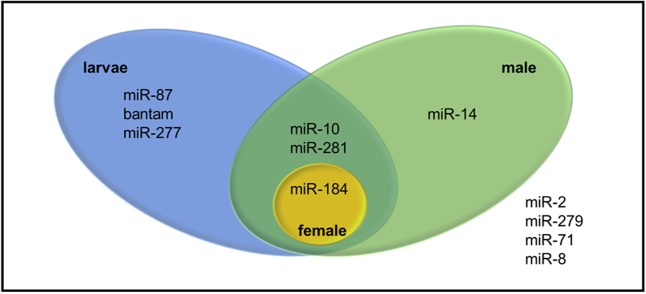
Venn diagram representing sample specific expression of WSS miRNAs. The four miRNAs listed outside the venn were identified from WSS samples other than pooled larva, male and female samples.

Pre-miRNA sequences were also examined in terms of the direction and the location on the transcriptome where one stem-loop might arise from different locations on the transcriptome. 38 transcripts were identified indeed as putative precursors of 18 mature miRNAs (**Supplementary Table S4**). While 22 of them stemmed from sense strand, 16 of them were found in antisense strand. Among putative miRNAs, only miRNAs from miR-184 and miR-281 families were identified from both sense and antisense strands. Since expression of the precursor transcripts in different WSS samples might reveal sample-specific miRNAs, all precursor transcripts were discriminated by the evidence of expression in larva and pooled adult WSS samples. Twelve mature miRNAs belonging to 7 miRNA families were identified in either larva, male, or female samples. Among them, only one mature miRNA was found in female as opposed to that of 9 mature miRNA sequences (4 miRNA families) in male and 10 mature miRNA sequences (6 miRNA families) in larva (**Figure 5**). The results showed that miR-184 was expressed in all three samples, whereas miR-14 was male-specific; and miR-87, bantam and miR-277 were larva-specific miRNAs. miR-10 and miR-281, on the other hand, were identified in both larva and male samples.

Further examination on sources of putative miRNAs suggested six lncRNA transcripts as putative precursors of miRNAs belonging to six miRNA families; miR-10, miR-14, miR-2, miR-279, miR-71, and miR-8. These lncRNAs were the only precursors identified for the respective miRNAs in WSS transcriptome. Among them, the lncRNA transcript, c46526_g1_i1, was identified as the precursor of miR-10 in both male and larva samples where c106582_g1_i1 was identified as the precursor of miR-14 in male sample only. Nevertheless, none of the lncRNA transcripts in female samples were identified as miRNA precursors. Expressions of remaining precursor lncRNA transcripts were detected in at least one of the remaining five WSS samples, supporting the expression of respective miRNAs at a sample specific level in WSS. These results also point out the functional importance of lncRNAs as being miRNA precursors.

### Putative Targets of WSS miRNAs

miRNAs regulate gene expression at the post-transcriptional level by interrupting expression through binding to the complementary sites on the target sequences. For 18 mature miRNAs, 32,149 and 6,458 miRNA-mRNA pairs were predicted using RNAhybrid and miRanda, respectively. A total of 5,070 unique mature miRNA-mRNA pairs, predicted by both algorithms were selected as reliable interaction pairs (**Supplementary Table S5**). From the larva miRNAs, miR-281 involved in the highest number of interactions with mRNAs (1,654), where bantam miRNA contributed in 70 interactions which was the lowest number between larval miRNAs (**Supplementary Table S5**). ∼282 mRNA targets were assigned per mature miRNA sequence on average. These large set of putative mRNA targets indicated the extend of the functional roles of miRNAs in WSS. Homology screenings against NCBI non-redundant (nr) protein database revealed sequence similarity of target mRNAs to the genes involved in several important MFs including binding, catalytic, molecular transducer, transporter and structural molecule. The most abundant term in biological process category was cellular and metabolic processes followed by biological regulation.

Other targets of putative mature miRNAs involved lncRNA transcripts. RNAhybrid predicted 20,788 mature miRNA-lncRNA pairs and miRanda predicted 1,075 miRNA-lncRNA pairs. 774 lncRNA transcripts suggesting 965 unique mature miRNA-lncRNA pairs predicted by the two algorithms (**Supplementary Table S5**). While the highest number of interactions was made by miR-184 within the larval miRNAs, miR-87 was involved in the least number of interactions with lncRNAs. ∼54 lncRNA targets were estimated per mature miRNA, indicating potential functions of lncRNAs as being miRNA targets although target mRNAs were shown to be more prevalent in WSS.

### lncRNA – miRNA – mRNA Network in WSS

**FIGURE 6:**
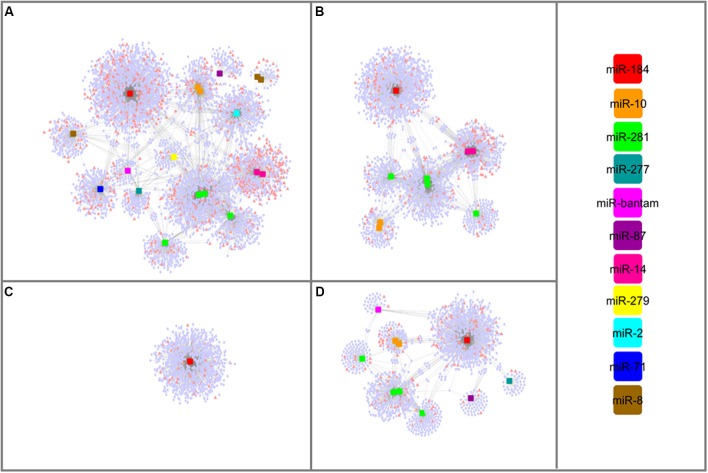
miRNA-mediated lncRNA and mRNA interaction networks. Networks constructed using; all WSS miRNAs **(A)**, male miRNAs **(B)**, female miRNAs **(C)** and larval miRNAs **(D)**. lncRNA transcripts were represented as pink triangles whereas pale blue circles were denoted to mRNA transcripts. miRNAs were shaped as squares and colored based on the color scale shown at right.

lncRNAs might involve in miRNA-mediated gene regulation through an indirect protection of target mRNAs, which called as target mimicry. By mimicking the binding site on the target mRNA sequence, lncRNAs might recruit miRNAs to enhance the expression of respective mRNAs. To have a broader aspect about these regulatory mechanisms, interaction networks between miRNA, lncRNA, and mRNAs were established combining miRNAs and their lncRNA and mRNA targets predicted here (**Supplementary Table S5**). Remarkable, all miRNA families had both mRNAs and lncRNAs as interacting partners. **Figure 6** illustrated that lncRNAs differentially expressed between larva and adult WSS samples were involved in one complex interaction network with miRNAs and mRNAs. All miRNAs identified from each growth stage of WSS contributed to the interaction network constructed in its respective stage. Functional annotation of mRNAs involved in any part of these networks was performed using Blast2GO to elucidate potential functions of lncRNAs as competing endogenous RNAs (ceRNAs). All mRNA and lncRNA targets of miRNAs were included in the combined network which build up one large and complex network. The results indicated that response to stimulus biological process was highly enriched in larva whereas structural molecule and transporter MFs in male. No enrichment was detected in female samples as all female miRNAs shared by larva and male. Overall, the interaction networks between miRNA, lncRNA, and mRNAs suggest putative roles of lncRNAs to increase regulation in variety of molecular processes through target mimicry for miRNAs.

### Bidirectional Mobility of miRNA in Wheat and WSS

Wheat Stem Sawfly larva accommodates in wheat stem and feeds from there until pupae stage of its life cycle (Delaney et al., 2010). Given the evidence of cross-kingdom regulation by miRNAs (Zhang et al., 2012; Tian et al., 2016; Jia et al., 2017), the interaction between intracellular molecules of WSS larvae and wheat cannot be underestimated due to these two organisms being in contact and trying to defeat each other. To assess possible effects of larval miRNAs on wheat gene expression and its response to WSS pathogen, target analysis for larval miRNAs was performed against *Triticum aestivum* coding sequences deposited at ensemble plant database using psRNAtarget tool. We identified 10 putative wheat targets for 3 miRNAs expressed at larvae. As shown in **Table 2**, a larva specific miRNA, miR-277, specifically targets several transcripts on the three sub-genomes of chromosome 3. Among the chromosome 3 targets, three transcripts were from chromosome 3B which was characterized with the wheat stem solidness (Nilsen et al., 2016). Blast screening of these chromosome 3 targets revealed similarity to methyltransferase PMT11 and ankyrin-like proteins (**Table 2**). Another larva specific miRNA, miR-87, has shown to have putative targets on chromosome 5BL and the only target for the male and larva shared miRNA, miR-281, was a transcript from chromosome 2AL of wheat. These 2A and 5B chromosomes were associated before with larval mortality. Although the predicted targets of miR-281 does not share homology with a protein with known function, targets of miR-87 was defined as vacuolar protein sorting-associated protein 22 homolog 1 (**Table 2**). Overall, these findings suggested that putative wheat targets of larval miRNAs were likely to be involved in defense mechanisms of wheat against insects.

**Table 2:** Wheat coding targets of WSS larval miRNAs.

Source	Mirna_Acc.	Wheat_target_Acc.	Target annotation
Larva	miR-277	TRIAE_CS42_3AL_TGACv1_193846_AA0620850.1	Methyltransferase PMT11 and ankyrin-like protein
Larva	miR-277	TRIAE_CS42_3AL_TGACv1_193846_AA0620850.2	Methyltransferase PMT11 and ankyrin-like protein
Larva	miR-277	TRIAE_CS42_3B_TGACv1_224524_AA0797630.1	Methyltransferase PMT11 and ankyrin-like protein
Larva	miR-277	TRIAE_CS42_3B_TGACv1_224524_AA0797630.2	Methyltransferase PMT11 and ankyrin-like protein
Larva	miR-277	TRIAE_CS42_3B_TGACv1_224524_AA0797630.3	Methyltransferase PMT11 and ankyrin-like protein
Larva	miR-277	TRIAE_CS42_3DL_TGACv1_251571_AA0882620.1	Methyltransferase PMT11 and ankyrin-like protein
Larva	miR-277	TRIAE_CS42_3DL_TGACv1_251571_AA0882620.2	Methyltransferase PMT11 and ankyrin-like protein
Larva	miR-87	TRIAE_CS42_5BL_TGACv1_408620_AA1363930.1	Vacuolar protein sorting-associated protein 22
Larva	miR-87	TRIAE_CS42_5BL_TGACv1_408620_AA1363930.2	Vacuolar protein sorting-associated protein 22
Larva, Male	miR-281	TRIAE_CS42_2AL_TGACv1_094608_AA0300450.1	Hypothetical protein F775_10692 [*Aegilops tauschii*]

miRNAs might pass from wheat to larva during their close contact. To assess putative larva targets, wheat mature miRNA sequences (119 entries) were retrieved from miRbase database. Using miRanda and RNAhybrid tools in combination, we identified 12,535 larval coding transcripts as putative targets of wheat miRNAs. The number of predicted targets varied widely between miRNAs, ranging from 2 to 6,174. Homology screening of the putative targets were performed based on blast hits from the NCBI non-redundant (nr) protein database with an *e*-value cutoff of 1E-5. Blast hits suggested that the genes targeted by wheat-derived miRNAs were likely to be involved in several functions such as kinases, helicases and transcription initiation factors. Among them, the two proteins with known functions targeted by more than 10 miRNAs were “Endothelin-converting enzyme 1-like isoform X1” and “*N*-acetylglucosamine-6-phosphate deacetylase.” Besides, digestive enzymes, i.e., lipases and glycogen synthases, were among the putative targets of wheat miRNAs.

## Discussion

Wheat production is severely limited by the a/biotic stress factors and biotic stress can account for up to 20% yield loss in wheat. Wheat Stem Sawfly (WSS; *C. cinctus* Norton) is the most harmful pest of wheat in North America (Beres et al., 2011), due to larval mining inside the plant stem. Although understanding their mechanisms of action is critical to fight effectively with WSS infestations and help farmers to reduce the devastation, very little is known on the genetic information and molecular mechanisms of WSS. To expand our knowledge, a detailed non-coding RNAs and their interactions with transcriptome has been conducted for WSS larvae and adults. Here we utilized a different method which is combining all reads from all tissues/samples. As many non-coding elements tend to show tissue specific expressions (Quinn and Chang, 2015), combining raw reads from different samples is important for the richness of the genetic elements available and the completeness of the transcriptome. Here, transcriptome-guided mRNA, lncRNA, and miRNA identification was performed with a focus on larvae transcriptomics and differential expression of transcripts between larvae and, female and male samples since most of the damage is caused from the larvae growing and feeding inside the wheat stem. Furthermore, the network between these RNA molecules besides the potential passage of WSS miRNA molecules toward wheat cells to target wheat coding sequences and to regulate the gene expression there as a part of its damaging effect has been disclosed.

With a stringent filtering of 165,284 transcripts in the *de novo* assembled WSS transcriptome, we identified 40,185 (24%) actively expressed protein-coding sequences. Of these transcripts, 1,251 transcripts were selected as novel mRNA candidates with lack of homology to known WSS mRNAs (**Supplementary Table S4**). To provide a broader aspect of their functions with non-coding RNA, these novel mRNA transcripts were classified in three GO categories, MF, biological process and cellular function. The functional annotations revealed proteins from many different molecular pathways, reflecting the complexity of eukaryotic cells. A significant number of these annotated proteins were ribosomal subunits, transcription and translation initiation factors, kinases, histone proteins which have important roles in the basic cellular mechanisms for the survival of the cell. In addition, six transcripts were identified as chemo-response-related proteins which might function in olfactory pathways that is important in sexual and social interactions of insects as discovered in honeybees (Benton, 2006; Pelosi et al., 2017). Another protein affecting insect behavior was longitudinals lacking (lola) protein which had three isoforms in WSS transcriptome assembly. This protein was found to be important in neuronal system development by maintaining proper axon guidance (Kuzin et al., 2005) and mutation studies in *D. melanogaster* resulted in aggressive behaviors on the insects (Edwards et al., 2006). These novel findings shed light on the undiscovered mechanisms in the cells of WSS and the organism being a social insect and reflected a potential to manipulate the developmental pathways of WSS in order to find more effective ways to cope with the infestations.

Dynamic changes in gene expression reflect the response of an organism to intrinsic and environmental signals. Thus, expression of genes varies over the course of a species’ life cycle; between stages of growth and development and between different sexual categories. Here, a total of 20,766 differentially expressed mRNAs were identified through pair-wise comparison of female and male samples to larva (**Supplementary Table S2**). Intriguingly, one fourth (6,525) of these transcripts showed sample specific expressions, indicating the distinct patterns of regulation between larva and adult developmental stages of WSS. While 6,019 of these differentially expressed transcripts were upregulated in larva when compared to adults, 14,824 of them were upregulated in adults, which could be a sign of a more complex cellular system in the adult stage of WSS life cycle. The cellular activity in larval stages of insect species was found to be less complex than it is in adults (Python and Stocker, 2002), which might have caused from the lack of complex behaviors in the larval stage while adult individuals are more motile and they involve in social interactions more often. The transcripts that showed a great differential expression between larva and both adult samples also emphasized the distinct cellular activities between larva and adults. Comparison of the expression levels of these transcripts in each sample revealed similar patterns of expression between male and female transcripts when compared to larva. **Figure 1** showed that transcripts upregulated in male compared to larva were also likely to be upregulated in female, although the level of regulation may differ. Intriguingly, most of these transcripts (16 out of 27) that showed the top 10 highest expression in one of the samples exhibited low-to-none expression (<2 fpkm) in any other samples, indicating the abundance of distinct regulatory mechanisms in different WSS life stages, thereby pointing out the functional importance of sample specific expressions of transcripts. We also included functional annotations of differentially expressed transcripts between larva and adult samples. Among them, allatostatin-A-receptor was one of the proteins that were encoded from the transcripts upregulated in larva. Allatostatin-A proteins were discovered to inhibit juvenile hormones in cockroach and cricket (Hergarden et al., 2012) which preserve the larval characteristics (Riddiford, 2012). Therefore, the upregulation of allatostatin-A-receptor might be a part of the passage through adult stage by contributing the inhibition of juvenile hormones. A number of transcripts upregulated in larva were encoding proteins related to circulatory system and central nervous system (CNS) development. Neurofibromin was one of the proteins annotated from two upregulated transcripts form larva, which was identified with its role in body size determination during larval development of *D. melanogaster* (Lee et al., 2013). In addition, chitinase was also encoded by 13 transcripts that were upregulated in larva. As an insect larva grows to form an adult individual, chitin molecules within the cuticle surrounding its body should be degraded by chitinases and synthesized again (Khajuria et al., 2010). Therefore, together with this information, it can be concluded that the cellular metabolism of the larva is focused on growth and formation of critical body systems leading to a complete adult development.

mRNAs are not the only players of molecular mechanisms where non-coding elements such as long non-coding RNAs (lncRNAs) were involved in various biological processes, including cell fate decision, developmental processes, sex-specific functions and growth (Keniry et al., 2012; Li et al., 2012; Militti et al., 2014). With the advent of high-throughput sequencing technologies, RNA-seq has boosted the identification and characterization of lncRNAs in several species. Despite the extensive studies on the functions of lncRNAs in *Drosophila* (Soshnev et al., 2011; Ecker et al., 2012; Li et al., 2012), little is known about characteristics and functions of lncRNAs in other flies (Xiao et al., 2015), including WSS. Major challenge in the identification of lncRNAs were that lncRNAs are not conserved between species. In fact, these are the non-conserved long transcripts that are not able to construct a full-length protein (Yan and Wang, 2012). A total of 59,676 novel lncRNAs were identified in this study that will likely be useful for further genomics research. Analysis for sample-specific expression profiles of lncRNAs showed the transcriptional diversity of lncRNAs between larva, female, and male WSS, supporting the evidence of the transcriptional diversity and specificity of lncRNAs in several species provided by recent studies (Cesana et al., 2011; Quinn and Chang, 2015). Interestingly, the results revealed that lncRNAs were much more abundant in larva than the adults (**Figure 3A**). This high abundancy of lncRNAs coincides with the high activity of developmental processes of larval stage of WSS life cycle. Thus, the results of this study supported the previous findings that the transcriptional diversity of lncRNAs could be related to developmental processes and sex-specific functions, even though further experiments are required to validate this conclusion. Notably, only 774 (1.3%) lncRNAs were common in all eight samples whereas 31,556 (52.9%) lncRNAs exhibited sample specific expressions. Thus, it is likely that a number of lncRNAs with tissue- or condition- specific expression exist and will be discovered through additional RNA-seq analyses at larger scales. In addition, the expression levels of lncRNAs are significantly lower when compared to the expression levels of protein-coding transcripts (Wu et al., 2014). The comparison of expression levels of WSS mRNAs and lncRNAs revealed that mRNAs from larva and adult stages were expressed relatively higher than the lncRNA molecules (**Figure 3B**), supporting the previous observations.

The major factor discriminating lncRNAs from mRNAs is lack of a discernable coding potential. Our tRNA analysis revealed that tRNA gene with anticodon CAT (tRNA-Met-CAT) decoding AUG start codon was found for both lncRNAs and mRNAs. Therefore, we identified that lncRNAs might do encode translation start codon, indicating the initiation of translation into proteins, as mRNAs do. 14% of tRNAs in lncRNAs corresponded to anticodon CAT (tRNA:Met-CAT) as opposed to that of 20% for mRNAs (**Figure 4**). With the highest abundance in each group, we could not correlate the initiation of translation with the potential of protein coding; however, distribution of remaining tRNA-anticodons differs broadly between mRNA and lncRNA transcripts. Content of the remaining tRNA species might regulate construction of the full-length and functional proteins.

Functions of lncRNAs can be inferred from their association with other non-coding elements. Several lncRNAs have shown to generate miRNAs, such as H19 lincRNA functioning as the precursor of miR-675 which in turn suppresses the growth promoting Insulin-like growth factor 1 receptor (*Igf1r*) (Keniry et al., 2012). Here, six lncRNA transcripts were identified as the only precursors of the six miRNAs; miR-10, miR-14, miR-2, miR279, miR-71 and miR-8. Besides being miRNA precursors, some lncRNAs act as miRNA targets. Through direct targeting, miRNAs might regulate the abundance of lncRNAs which are involved in different cell functions (Yoon et al., 2014). Several lncRNAs targeted by miRNAs have been uncovered recently, such as lincRNA-p21 (Yoon et al., 2012) and H19 (Kallen et al., 2013). The assessment of the possible miRNA-lncRNA target interactions identified 54 putative lncRNA targets per miRNA agents. Having the miRNA binding site, lncRNAs might enhance the functioning of miRNA target genes by titrating shared miRNAs from environment. As lncRNAs targeted by miRNAs could be involved in a regulatory circuitry between lncRNAs, miRNAs and mRNAs, we investigated putative target mimicry functions of these lncRNAs. The first evidences of target mimicry were discovered in plants (Franco-Zorrilla et al., 2007). Later, several examples were identified in mammals in the name of competing endogenous RNAs (ceRNAs) of miRNA targets (Cesana et al., 2011; Karreth et al., 2011; Kallen et al., 2013). Here, we also constructed a putative interaction network between lncRNAs, miRNAs, and mRNAs in WSS to identify putative lncRNAs acting as ceRNAs (**Figure 6**). Experimental validation of target lncRNAs might shed light of the regulatory functions of these networks. We believe that importance of lncRNAs and such regulatory networks will emerge further.

The journey through understanding the functions of miRNAs has started with the discovery of lin-4 and its role in larval development in *C. elegans*. Lin-4 miRNA was upregulated in *C. elegans* larvae in one of the four larval stages, targeting lin-14 mRNA, suggesting that it has a regulatory role in larval development (He and Hannon, 2004; Alvarez-Garcia and Miska, 2005). The importance of miRNAs in developmental-timing of larvae was also shown in vertebrates in several studies. Here, we identified three miRNAs specifically expressed at larval stages of WSS; miR-87, bantam and miR-277. miR-87 was suggested as a regulator of the immune responses of mosquitoes against viral infections (Liu et al., 2015). Later, its expression was identified in the nematode, *Meloidogyne incognita* (Zhang et al., 2016); however, its function in insects remains elusive. Functions of putative targets of miR-87 includes transferase activity, topoisomerase activity, binding and extracellular matrix structural constituent, suggesting its structural and functional importance. Both of miR-277 and bantam miRNA were associated with anti-apoptotic activities in insects (Jones et al., 2013; Bilak et al., 2014). Although direct targets and function of miR-277 requires further evidence, miRNA bantam was linked directly to protective functions ensuring cell proliferation (Bilak et al., 2014). As the larval stages of WSS are the most stressed periods in WSS stages, increased regulation through bantam miRNA and miR-277 in the larva samples supported its anti-apoptotic activities. On the other hand, miR-14, the only adult male-specific miRNA identified is expressed greatly in testicular tissues of immature and fully-mature adult *Bactrocera dorsalis* flies, and its target was putatively identified as β2-tubulin (Tariq et al., 2015). The function of β2-tubulin was first revealed in *D. melanogaster* as maintaining the mobility of sperms (Zimowska et al., 2009). These findings support the idea that miR-14 is a male-specific miRNA functioning in WSS adult male testes.

Plants have evolved mechanisms to protect themselves from herbivorous feeding. In the case of an insect attack, defense mechanisms in plants are triggered by signals such as touch, oviposition, tissue damage and molecules coming from the insect (Chung et al., 2013). On the other hand, insects use effector molecules to suppress or manipulate defense response in host plant (Hogenhout and Bos, 2011; Erb et al., 2012). For example, a recent study showed that small RNA molecules of a fungi species, *Botrytis cinerea*, inhibiting the RNAi machinery and silencing the genes for plant immunity through binding to AGO1 protein of its host plant Arabidopsis (Weiberg et al., 2013). Another study showed that host target sequences of *Phytophthora parasitica* sRNAs were transcribed at the low or undetectable levels (Jia et al., 2017). In the light of these findings, we considered larval miRNAs affecting host wheat plants to regulate gene expression in favor of larval survival. Target prediction analysis of larvae miRNAs brought out the possible interactions with wheat protein-coding sequences, which may result in the blockage of resistance to larval feeding. Intriguingly, miR-277 was shown to target several loci on chromosome 3B, which has been associated with the stem solidness feature of wheat. Predicted wheat targets of these transcripts showed significant similarity to methyltransferases and Ankyrin-like proteins. Ankyrin, a repeat domain, is important for several protein–protein interactions (Becerra et al., 2004). One of the best-studied functions of Ankyrin-like proteins is pathogen resistance through regulation of salicylic acid-induced gene expression (Despres et al., 2000; Lu et al., 2003). Thus, it is tempting to speculate on the interactions between larval miR-277 and plant RNAs, potentially affecting stem solidness and plant defense, thus decreasing resistance to larval feeding inside the stem.

Since WSS larvae eat plant tissues for survival, it is very likely that plant miRNAs are taken inside of the insect body within their dietary consumptions. Several studies have provided evidence of *trans*-kingdom transfer of sRNAs from plant to other species which are in close contact; plant to virion (Iqbal et al., 2017), plant to nematodes (Tian et al., 2016), and plant to animal during feeding (Zhang et al., 2012). Wheat miRNAs might also act as the regulators of insect metabolism. Here, we showed potential larval targets of wheat miRNAs. However, these initial findings are needed to be validated to conclude on cross-kingdom miRNA regulation between WSS and wheat species.

## Author Contributions

HB concieved and designed the study and supervised all the analysis and drafted manuscript. SB and HC performed the analysis and drafted manuscript.

## Conflict of Interest Statement

The authors declare that the research was conducted in the absence of any commercial or financial relationships that could be construed as a potential conflict of interest.

## Supplementary Material

FIGURE S1 Correlation between length and GC content in lncRNA and mRNA transcripts. **(A)** GC content distribution of mRNA and lncRNA transcripts. **(B,C)** Association between length and GC content in lncRNA and mRNA transcripts.

TABLE S1 Sequence Read Archieve (SRA) run table.

TABLE S2 Homology screenings of all WSS mRNAs.

TABLE S3 Annotations of novel mRNAs.

TABLE S4 List of putative miRNAs identified in the assembly of WSS transcriptome.

TABLE S5 miRNA-lncRNA target pairs utilized in lncRNA-miRNA-mRNA networks.

DATA S1 Wheat Stem Sawfly (WSS) mRNA sequences in fasta format. Available at: https://drive.google.com/open?id=0B_T-7LzZ58ySbW1Uc1ppUnRKalE.

DATA S2 Wheat Stem Sawfly lncRNA sequences in fasta format. Available at: https://drive.google.com/open?id=0B_T-7LzZ58ySbW1Uc1ppUnRKalE.
