# Supplementary figures and images for: Assembly and Annotation of Transcriptome Provided Evidence of miRNA Mobility between Wheat and Wheat Stem Sawfly
Source: Front Plant Sci. 2017 Sep 26;8:1653. doi: 10.3389/fpls.2017.01653 (PMC5630980; doi:10.3389/fpls.2017.01653)

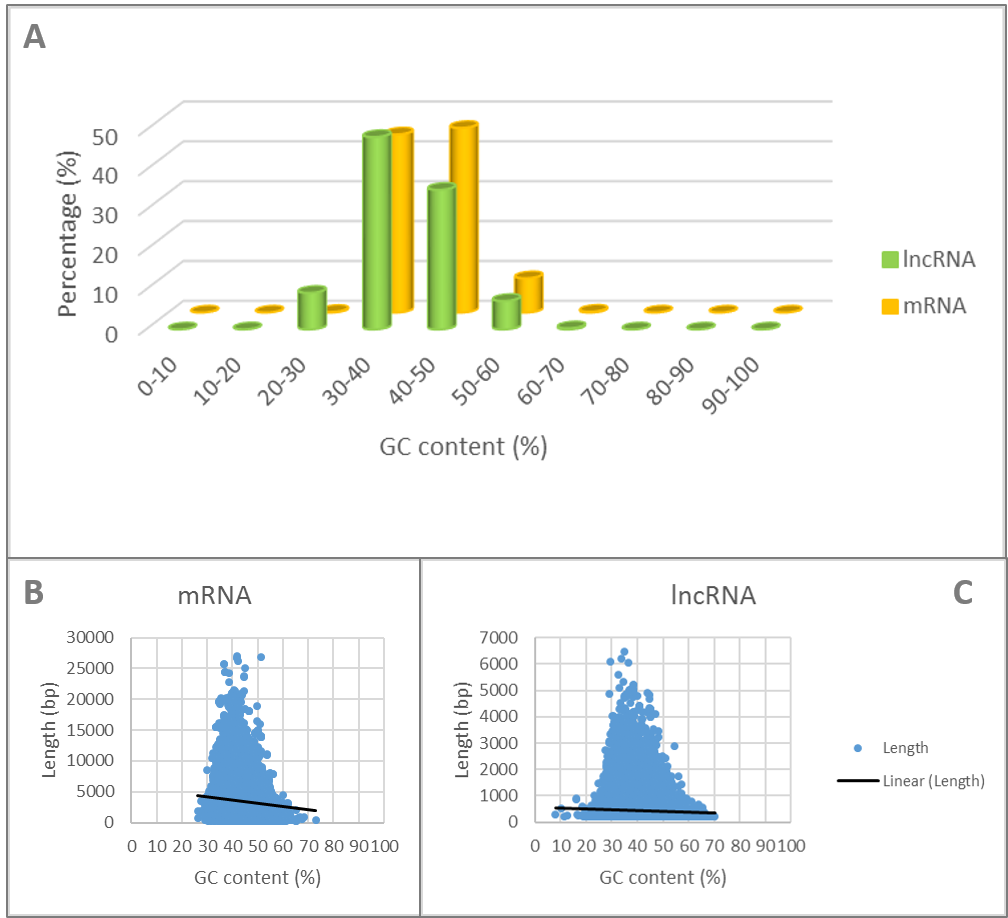

Supplement: FIGURE S1 — Correlation between length and GC content in lncRNA and mRNA transcripts. (A) GC content distribution of mRNA and lncRNA transcripts. (B,C) Association between length and GC content in lncRNA and mRNA transcripts. [file Image_1.TIF]
